# Gaston Ramon’s Big Four

**DOI:** 10.3390/toxins16010033

**Published:** 2024-01-09

**Authors:** Jean-Philippe Chippaux

**Affiliations:** MERIT, Institut de Recherche pour le Développement, Université Paris Cité, F-75006 Paris, France; jean-philippe.chippaux@ird.fr

## 1. Introduction

When immunology was still in its infancy, Gaston Ramon made several major contributions to humoral immunology. These complementary discoveries were all published in a short period between the two world wars, specifically between 1922 and 1926, and were followed by 810 of the 867 articles Ramon published in total (https://bibnum.pasteur.fr/app/photopro.sk/pasteur/?#biblio/31726, accessed on 22 December 2023). Ramon spent the rest of his life developing applications and improvements for the prevention and treatment of numerous infectious diseases.

The modern concept of immunology began with Jenner’s smallpox vaccination. It is based on the vaccines developed by Louis Pasteur in eight successive periods corresponding to the technological innovations: (1) attenuation of living pathogens, following in the footsteps of Jenner and Pasteur, (2) inactivation of pathogens after killing them, described by Salmon at the end of the 19th century, (3) the use of Ramon’s toxoids from 1922, (4) optimization of the humoral immune response with adjuvants, also resulting from Ramon’s work in 1924, (5) the combination of antigens in 1926, (6) the cell culture of viruses initiated in the 1950s, enabling the large-scale production of antigens and reassortment of viral genetic material by co-infection, (7) the contributions of genetic engineering after 1965, and (8) methods of stimulating the cellular immune response required for immune memory and stable, long-lasting immunity since the late 1970s. The combination of these different contributions to active immunization has made it possible to develop increasingly effective vaccines, including against inert or unstable antigens or pathogens of low immunogenicity, such as certain parasites or viruses that escape the adaptive immune response. Other advances include vaccines using nucleic acids instead of proteins, new routes of administration, proteomics and “reverse vaccinology” (the identification and use of genes coding for protective proteins), the validation of strategies concerning vaccine administration, targets (children, adolescents, women of childbearing age, elderly subjects, patients with comorbidities, etc.) and appropriate logistics depending on whether the vaccine is preventative or therapeutic, with a view to personalized vaccination [1].

Serotherapy, or passive immunotherapy, involves transferring specific antibodies from a previously immunized subject to a naïve individual for prophylactic or curative purposes. The first step is to immunize an animal to produce specific antibodies. The antibodies are sampled, purified and stored until they can be used in an appropriate way. Serotherapy has evolved in parallel with the history of vaccination, or active immunization, which involves producing one’s own antibodies [2].

In a series of articles, Héricourt (1850–1938) and Richet (1850–1935) had described the specific immunity developed by a dog infected with a staphylococcus (probably a strain of *S. aureus* according to Lahaie and Watier [3]) and the transfer of this “resistance” to rabbits after the intraperitoneal inoculation of blood taken from the dog [4,5]. Behring (1854–1917) and Kitasato (1853–1931), who disputed the validity of these results, immunized rabbits against tetanus and inoculated their serum into mice, which resisted injection of a lethal dose of toxin from a *Clostridium tetani* culture [6]. A week after this paper, Behring reported identical results, this time against diphtheria in guinea pigs, murids being resistant to diphtheria [7]. The main difference between the experiments of Héricourt and Richet, and those of Behring and Kitasato lies in the latter’s use of serum instead of whole blood, which is toxic via the venous route but well tolerated through the intraperitoneal route [5]. Bouchard demonstrated that serum contained an essential part of the protective factors, but that blood cells also played a role (apud [8]). Behring and Ehrlich (1854–1915) theorized the concepts of passive immunity and humoral immunity, contrasting the latter with the cellular immunity formalized by Metchnikoff (1845–1916). Ehrlich was the first to introduce the notion of the antibody (=antikörper), a protein substance present in serum and responsible for specific protection [9].

Serotherapy rapidly developed as a preventative (rabies, tetanus) or curative (tetanus, diphtheria) therapy, and was widely used until the Second World War. By contrast, vaccination, particularly against diphtheria and tetanus, was long delayed due to the toxic effects of the toxin used as an immunogen.

Gaston Ramon’s personal and professional career was decisive (Box 1). His work at the Institut Pasteur enabled him to observe phenomena that were commonplace but essential to in understanding immunization against infectious agents. He knew how to use them to interpret the phenomena he studied.

Box 1 **Gaston Ramon biography (The author lives in Bellechaume and was able to gather original information about Gaston Ramon) [10,11,12].**

**Figure 1:**
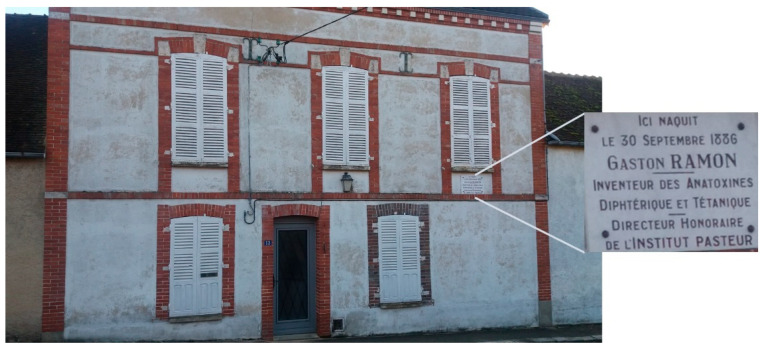
The birthplace of Gaston Ramon in Bellechaume. “Gaston Ramon was born here on September 30, 1886. Inventor of diphtheria and tetanus toxoids. Honorary Director of the Pasteur Institute” (photo by JP Chippaux).

 Gaston Ramon was born on 30 September 1886 in Bellechaume, a small village in Burgundy (Figure 1). His father was a baker. At the age of 4, the family moved to the nearby town of Sens. There, young Gaston attended primary and secondary school.

 After passing his baccalaureate, Gaston Ramon spent a year preparing for the veterinary school entrance exam, which he passed in 1905 with a rank that enabled him to choose the prestigious veterinary school at Maisons-Alfort, near Paris. However, before entering the veterinary school, Gaston Ramon carried out his military service with the 30th artillery regiment in Orléans. He joined the Maisons-Alfort school in 1906, where his main subjects were chemistry and infectious diseases. His head of practical work, Alexandre Augustin Monvoisin, taught him that a few milliliters of formaldehyde prevented milk from turning, without altering its composition or properties. He also learned photography techniques, notably the photographic lantern and autochrome, that were recently discovered by the Lumière brothers. This training would prove particularly useful in his future discoveries.

 He graduated from the veterinary school in 1910. After a few months with the Seine veterinary health service, in charge of animal disease surveillance and epizootic control, as well as the sanitary inspection of markets, slaughterhouses and meat safety, he was introduced to Emile Roux, Director of the Institut Pasteur. Roux recruited him as deputy head of the therapeutic serum preparation department at the Garches facility on the outskirts of Paris. Gaston Ramon was responsible for immunizing several hundred horses, collecting their plasma, checking the neutralizing power of the serums, treating the horses and ensuring their good health. The First World War revealed Gaston Ramon’s organizational skills. With the enemy closing in on Paris, Gaston Ramon had to transfer the serotherapy department to Toulouse in the south of France, but above all he had to meet the explosion in demand for serums for both civilian (diphtheria antitoxin) and military needs (tetanus antitoxin and antigangrenous serum in particular). In 1917, he married Marthe Moment, grand-niece and god-daughter of Emile Roux.

 However, his ambition was to become involved in scientific research, which he was able to do from 1920, while continuing his serotherapy production activities. Shortly after the Armistice, he was finally able to carry out his own research in a small laboratory he had set up in Garches, near his home. It was here that he made the discoveries that made him famous. In 1926, he took over the management of the Garches facility. Building on his success and international renown, he developed a research center with several laboratories, notably in biochemistry and cellular pathology.

 When Emile Roux died in 1933, he was replaced by Louis Martin, and Gaston Ramon became deputy director. He succeeded Louis Martin in 1940 when the latter retired. However, discouraged by the opposition preventing him from realizing his ambitious plans, he resigned 6 months later. He returned to the Garches facility to resume serum and vaccine production. Caught up in the turmoil of the Second World War, Gaston Ramon was torn between maintaining the production of serums and vaccines essential to public health—including meeting the German army’s requests for serums—and his patriotism, one expression of which was his submission to Marshal Philippe Pétain. In 1944, the Comité d’épuration (purge committee), responsible for removing Institut Pasteur researchers convicted of collaborating with the enemy, released Gaston Ramon. However, it was decided to remove him from the management of the Garches facility, enabling Institut Pasteur to regain direct control at a time when its finances were in difficulty.

 Gaston Ramon left the Institut Pasteur in 1948. In 1949, he was unanimously elected Director of the Office International des Epizooties by representatives of 40 nations. He retired in 1959. He was a member of several national academies (veterinary, medicine, and science) and ‘honoris causa’ doctor of various universities.

 He died on June 8, 1963 and is buried in Bellechaume.

Here, we review the circumstances, importance, and fate of Ramon’s main discoveries: (a) the initial flocculation reaction in 1922 [13], (b) the principle of toxoids and their vaccination in 1923–1925 [14,15,16], (c) the role of adjuvant and immunostimulant substances in immunization in 1925 [17,18], and (d) its corollary, associated vaccinations from 1926 [19].

## 2. The Early Beginnings of Ramon’s Discoveries

Having learned during his veterinary training that the quantity of formalin contained in a thimble (around 2 mL) is enough to prevent a liter of milk from turning without altering its taste and properties, Gaston Ramon was able to exploit this characteristic on several occasions in his career [11]. Similarly, during his routine activities in the serotherapy department of the Garches annex, he scrupulously noted the details of his manipulations, from which he was to derive many observations that would later prove very useful.

Throughout World War I, Gaston Ramon managed the Institut Pasteur’s anti-infectious serum production. In 1914, 233 horses provided 80,000 monthly doses. The stable soon welcomed 1462 horses, bringing the number of monthly doses to over 150,000, mainly intended for allied combatants. During the German spring offensive (March–April 1918), up to 20,000 vials of anti-tetanus serum were delivered daily to the army health service, in addition to other serums (against diphtheria, meningococcus, gangrene, dysentery, plague), vaccines (rabies, typhoid, plague) and veterinary products for horses, which were still widely used on the battlefield (notably mallein against glanders and a vaccine against epidemic lymphangitis) [20]. The systematic use of anti-tetanus serum among the wounded, thanks to increased supplies and the gradual formalization of its indication in the various allied armies, reduced tetanus morbidity from 8‰ to less than 0.2‰ between the beginning and end of the conflict [21,22].

In 1915, Roux asked Ramon to find a technique for sterilizing therapeutic serums that were frequently contaminated during manufacture or storage, causing abscesses in treated patients. Ramon came up with the idea of adding formalin to the serum at a concentration of 1‰, achieving a mixture that was as clear, effective and well-tolerated as it was devoid of infectious risk. This technique was subsequently improved by heating the mixture for 1 h at 55 °C [23].

During horse immunization, Ramon observed that those horses producing antitoxin-rich serum developed abscesses whose size was proportional to the antitoxin titer of the serum [17]. This observation was later confirmed by the in vitro titration developed by Ramon (see below).

## 3. First Discovery: The Flocculation Mechanism (1922)

The formation of a precipitate when mixing an antigen (e.g., a pathogen in culture) with serum from animals repeatedly inoculated with the same antigen had been observed since the end of the 19th century. However, this precipitate was not constant and its causes and circumstances of occurrence were not clearly identified [24,25].

Until 1922, the titration of the antitoxic power of sera was carried out by in vivo neutralization tests in guinea pigs and sometimes in mice, depending on the sensitivity of these species to the antigen. As early as 1920, to replace these slow, costly and error-prone tests, Ramon studied the formation of the toxin–antitoxin complex. He found that the precipitate formed was a function of the relative amounts of the two compounds. These properties have been the subject of numerous fundamental and practical studies: the in vitro titration of sera, the evaluation of the antigenic power of toxins and anatoxins, immunogenicity of antibodies, study of the antigen-antibody complex, purification of the toxin and use of the complex as a vaccine.

The “initial flocculation” described by Ramon—later called the “antigen-antibody complex”—reflects a close interaction between the two molecules. Knowing the amount of one of the two molecules in the mixture allows us to deduce the amount of the other molecule and therefore its titer [26]. By mixing variable dilutions of antitoxin with a fixed amount of toxin, we can observe the initial flocculation of one of the dilutions, which corresponds to the antitoxin titer of the serum. The strength and rapidity of the flocculation are greatest when the diphtheria toxin and the anti-diphtheria serum are exactly in equilibrium. Ramon demonstrated the specific character of antitoxins prepared against a toxin, regardless of the animal immunized or the immunization technique used [27,28]. Thus, he was able to use the flocculation technique—the results of which correlated perfectly with Ehrlich’s in vivo assay technique in use at the time—to titrate the diphtheria antitoxin he was producing [29,30]. The reaction was accelerated by performing it at 45 °C, although its accuracy was greater at laboratory temperatures, where it was clearly distinguishable from secondary flocculations that can be considered artifacts [31]. Ramon noted that the rate of flocculation varied from horse to horse, but was independent of the type of antigen (native toxin, toxoid, or cultured bacteria) and the titer of the antitoxin. He suggested that the rate of flocculation is related to the horse’s state of specific immunity prior to the start of immunization, which is also true for humans. According to Ramon, two characteristics support the quality of the antitoxin: one is quantitative, the serum titer, and the other is qualitative, the flocculation rate. He hypothesized that natural immunity would be strengthened by repeated injections of antigen, suggesting the principle of immune memory and the booster effect [28,32,33]. Analyses of the antigen–antibody complex quickly revealed the organization of the binding, and particularly the valences of the antibodies, i.e., their affinity for structurally similar proteins [34]. Later, this led to the principle of antibody paraspecificity and cross-reactivity.

The concept of flocculation has led to the discovery of numerous applications, including precipitation reactions used for diagnosis, assay or functional studies of active proteins. Tiselius and Kabat demonstrated via electrophoresis of horse and rabbit serum immunized against type I pneumococcus that the antibodies belonged to the gamma globulins, opening the way for the study of antibody structure and function, as well as to the notion of epitopes, which largely explain the mechanisms of antibody function [35,36,37]. By precipitating the antigen–antibody complex in a gel, Ouchterlony enabled the development of many diagnostic techniques [38,39]. Immunoelectrophoresis relies on two distinct properties of proteins: electrical charge and immunological affinity between antigen and antibody. It can also be used to check the purity of a protein or the functional activity of an enzyme when an appropriate substrate is added to the gel [39,40].

The development of immunoassays has shown that the affinity of an antibody for the antigen that led to its production does not systematically correlate with its neutralizing capacity. Long considered a curiosity, the properties of non-neutralizing antibodies—also known as binding antibodies—have been little studied. As antigen-specific markers, they are useful for diagnostic testing and epidemiological surveys, but of less importance for pathogen control. The contribution of non-neutralizing antibodies to specific immunity and their use in reducing viral replication, activating defense mechanisms or blocking the intracellular viral cycle are beginning to be better understood [41,42].

## 4. Second Discovery: Toxoids (1923)

The discovery of toxoids was also the result of specific observation and research. During the development of the flocculation reaction, diphtheria and tetanus toxins were produced from broth cultures kept at different temperatures—from the laboratory temperature to that of the oven (38 °C)—which increased the risk of contamination and made it difficult to evaluate the results. During the development of the flocculation reaction, diphtheria and tetanus toxins were produced from broth cultures stored at different temperatures—from laboratory to oven (38 °C)—increasing the risk of contamination and making it difficult to evaluate results [23]. Ramon had the idea of sterilizing the preparation with formalin, which he found did not prevent the flocculation reaction. He concluded that the broth was protected from bacterial contamination and that the toxin had not lost its ability to be recognized by antibodies [43]. In this preliminary study, Ramon found that the toxicity of the toxin gradually decreased until it disappeared, without reducing the flocculation power, which remained very stable. Ramon then demonstrated that formalin combined with heat eliminated the toxicity of the toxin without altering its antigenicity or immunogenicity, thus introducing the toxoid principle [11]. In the following years, he developed this paradigm and extended it to natural toxins—bacterial and animal—and plant extracts—plant toxins and alkaloids—then to viruses and toxins. The preservation of the immunizing properties, which is the strength of Ramon’s “anatoxine”, is the main difference with Ehrlich’s “toxoid” which he himself defined as “a degenerate form of toxin, in which the toxiphor group has been affected, the combining power has been maintained but the power to produce antitoxin or immunity has been lost” [15,44,45,46]. During his lifetime, Ramon made a point of distinguishing between Ehrlich’s concept of toxoid (the term “anatoxine” is composed of the Greek prefix “ana-” which means “opposite” to mark the loss of toxicity of the toxin and is distinguished from the prefix “a-” which simply expresses the absence of toxicity [15]) and that of “anatoxine” because of this difference, which he rightly considered fundamental [15]. On the other hand, Ehrlich deserves credit for showing that toxic and antigenic potency correspond to two different sites of the toxin [46].

Moreover, the toxicity of diphtheria and tetanus toxins prevented their use as vaccines in humans. After the discovery of the toxin and its properties, several attempts were made to reduce the toxicity. Iode trichloride [7], thymus extracts [47], heating of the culture medium [48] or the action of iodine [49] allowed the immunization of animals to produce therapeutic sera, but proved insufficient to vaccinate humans. 

The toxin–antitoxin flocculate was first proposed for immunization against diphtheria toxin [50]. However, the anatoxin–antitoxin flocculate, which was equally immunogenic, presented less risk of toxicity because the anatoxin replacing the toxin was non-toxic [51,52]. Ramon would soon show that the anatoxin alone was easier to prepare and more effective, paving the way for active immunization against the diphtheria toxin and preventative vaccination against diphtheria [53].

The preparation of numerous toxoids—as many vaccines—would be developed in the following decades. However, diphtheria and tetanus were the main beneficiaries.

Until the end of the 19th century, diphtheria—long known as “croup”—wreaked havoc among children and, to a lesser extent, adults. In Paris, mortality between 1865 and 1893 averaged 1500 deaths per year, or about 100 deaths per 100,000 inhabitants [54]. The symptoms of diphtheria were fully reproduced in animals injected with the culture broth in which the bacteria had developed and from which it had been removed [55]. Similarly, the pathogenesis of tetanus was linked to the toxin produced by *Clostridium tetani* [56]. At the same time, Behring and Kitasato, who had immunized rabbits against tetanus, inoculated their serum into mice that resisted injection with a lethal dose of toxin from a *Clostridium tetani* culture, describing passive immunotherapy or serotherapy [6].

The management of patients with diphtheria or tetanus became more widespread after 1894, especially in urban areas, leading to a reduction in the lethality of both diseases. By 1923, the incidence of diphtheria had not changed, but the case fatality rate had dropped from over 50% to about 10% [57].

Vaccination was introduced in the French armed forces in 1930 and became compulsory in 1932. Mandatory vaccination of all children, decreed in 1938, took more than a decade to implement due to the war and the actions of anti-vaccinationists. While the average annual morbidity before 1945 was over 45,000 cases, including 3000 deaths, it fell below 3000 cases in 1951, to 1000 cases in 1960 including 36 deaths, and then to 50 cases in 1970 including 3 deaths [57,58]. Since 1980, the average incidence has been 4.6 cases per year, with vaccination coverage of 95.7% [59,60].

In the USA, 200,000 cases of diphtheria per year (140 to 150 cases per 100,000 population) with up to 15,000 deaths were reported before routine vaccination was introduced. Vaccination spread after 1940, and high vaccination coverage reduced morbidity to 19,000 in 1945 (15 cases per 100,000 population). From 1980, morbidity became negligible (1.4 cases per year on average) thanks to 94% vaccination coverage (for the three doses of the trivalent diphtheria–tetanus–pertussis vaccine) [60,61].

In the French army, whose vaccination coverage in 1940 is difficult to assess because of the large number of people mobilized, it was not possible to evaluate the benefits of tetanus vaccination. The number of casualties was small and, above all, the health service was rapidly disorganized and unable to register tetanus cases.

After some hesitation, tetanus vaccination was introduced in the British Army in 1939. Since vaccination was not mandatory in Great Britain, serotherapy was used in the event of injury. Vaccination coverage was estimated to be 90% at the beginning of the war and nearly 100% by the end. The incidence rate of open-wound tetanus, which had been 1.47‰ during World War I, fell to 0.12‰ during World War II [62].

Beginning in 1941, tetanus vaccination became widespread in the U.S. Army, with excellent coverage. For open wounds, a tetanus toxoid booster was given instead of serotherapy. This strategy limited the number of tetanus cases during World War II to three, only one of which occurred in combat [63]. At the end of the war, the Garches hospital on the outskirts of Paris treated many wounded Americans and a few Germans. While none of the Americans—all of whom had been vaccinated—contracted tetanus, 20 German soldiers did, and 12 died [64]. However, the total number of American and German soldiers treated at the Garches hospital has not been released.

## 5. Third Discovery: Adjuvant (1925)

Ramon defines an adjuvant as “a specifically inert substance which, when injected in association with the specific vaccine antigen, increases to a greater or lesser extent the immunity that the latter is capable of developing” or, more simply, “an inert substance which, when combined with an antigen, induces an immune response superior to that of the antigen administered alone” [18,65]. According to Dresser, a conventional adjuvant is a substance that increases the antibody titer against an antigen. Adjuvanticity, on the other hand, describes the property of an antigen (intrinsic adjuvanticity) or of a substance added to the antigen (extrinsic adjuvanticity) that induces specific immunity against the antigen without presupposing its mode of action or immunological effect [66,67,68,69].

There are many potential adjuvants—although very few are used in human medicine—and different classifications based on their source (botanical, bacterial, chemical, or hormonal), molecular structure (particulate, non-particulate, etc.), target, mode of action, use, etc. [70,71,72,73].

### 5.1. History of Adjuvants

As early as 1913, during the production of antitoxin, Ramon observed abscess formation at the site of antigen injection and a higher yield in horses with a strong local inflammatory reaction [17,74]. This phenomenon was demonstrated, explained and exploited in the development of the flocculation reaction. In vivo titration of antitoxin did not allow the measurement of the individual yield of antitoxin production by horses. For economic reasons, the in vivo titration was performed on pools of sera. Secondly, for technical reasons, it took a long time to obtain the results. In vitro flocculation titration eliminated both limitations.

Ramon deduced that the kinetics of antibody production in immunized animals (initial rise in antibody levels followed by a plateau phase) was due to local inflammation at the site of antigen injection, and performed experiments to prove it [17,75]. It soon became apparent that it was not necessary to induce an abscess, but that a simple local inflammatory edema with leukocyte influx could substantially increase antitoxin production. This was the origin of the depot effect theory, which is part of the basis of adjuvanticity [65]. Ramon tested numerous substances (pus or germs from an abscess in another horse, calcium chloride, glycerin, gelatin, agar, insoluble serum, bread crumbs, aleurone, potato starch, milk, lecithin, lanolin, cholesterol, petroleum jelly, atoxyl, rubber latex, tannin, pectin [65]). Starches—especially tapioca—initially presented the best profile. On the one hand, they adsorbed the antigen, forming a complex that could be easily manipulated, and on the other hand, they induced a mild local irritation that gradually resorbed without leaving a trace, while gradually releasing the antigen [18,76]. It was subsequently replaced by other adjuvants chosen first empirically and then based on immunological discoveries [77,78].

As early as 1926, aluminum salts, especially alum (=potassium aluminum sulfate), showed great promise [51]. Their efficacy was attributed to their ability to adsorb and gradually release antigens [79]. For a long time, aluminum salts were the only extrinsic adjuvants, i.e., not contained in the antigen, used in human clinical practice.

**Figure 2:**
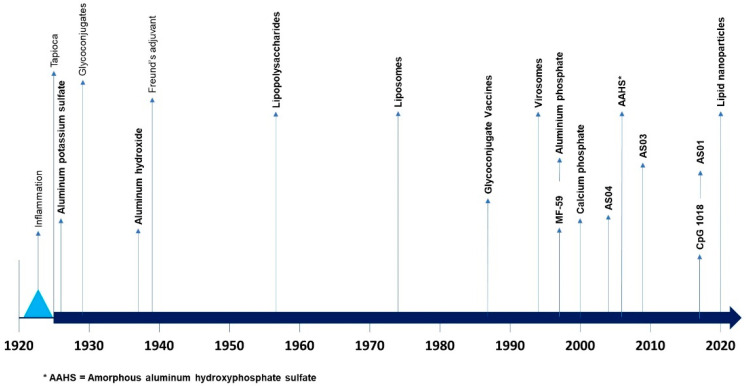
Chronology of major events regarding adjuvants (from [78,82]; * AAHS = Amorphous aluminium hydroxyphosphate sulfate.).

Subsequently, numerous substances were tested, both to obtain new, more effective, and better tolerated adjuvants, and to understand their mode of action and effects on immunity. Only a few of these have been used in animals or humans (Figure 2).

Adjuvants, alone or in combination with a candidate vaccine antigen, undergo rigorous toxicological, pharmacological and clinical evaluation to ensure their safety and tolerance [80,81].

### 5.2. Mode of Action of Adjuvants

The role of the adjuvant is not limited to the quantitative and sustained production of antibodies. The appropriate immunological response, depending on the objective pursued, is based on the optimization of the humoral and cellular immune response, the enhancement of antigen presentation, the stimulation of non-specific immunity and the qualitative modalities of the response obtained, which are the focus of the current research [81,83].

**Figure 3:**
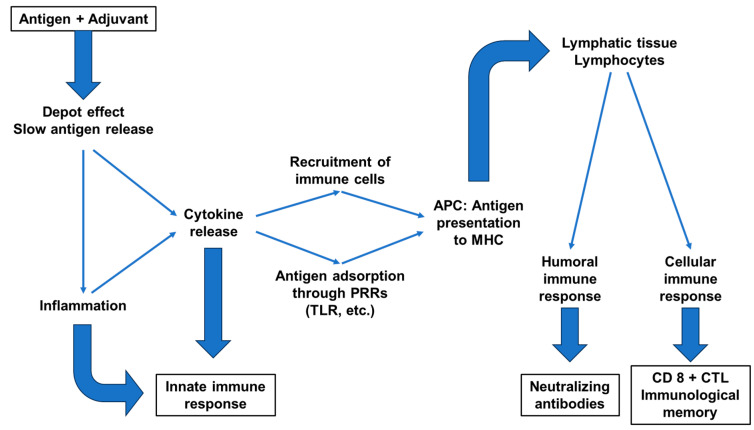
Effects of adjuvants on the immune response (from [88,89]); (APC = antigen-presenting cell; PRR = pattern recognition receptor; TLR = toll-like receptor; MHC = major histocompatibility complex; CTL = cytotoxic T lymphocyte).

The general action of adjuvants includes the stabilization of antigenic epitopes, retention of the antigen at the vaccine injection site (depot effect), optimization of antigen uptake by antigen presenting cells (APCs) for improved presentation to the lymphatic system and the stimulation of humoral and cellular immune pathways (Figure 3) [71].

Based on their mechanisms of action, adjuvants can be classified as delivery systems and immunostimulants [78]. These modes of action are potentially complementary and are increasingly combined in adjuvant formulations of varying complexity (Figure 3; Table 1).

Adjuvants involved in delivery systems facilitate antigen transport by APCs to optimize recognition, identification and degradation by major histocompatibility complexes (MHCs), which are central to both humoral and cellular adaptive immune responses [84,85,86].

Immunostimulants can be defined by (a) their chemical nature or structure, corresponding to the different categories of adjuvants, (b) their mode of action, or (c) the cellular and molecular effect they induce. Ultimately, what they have in common is the pronounced improvement in the observed humoral and/or cellular immune response [87].

Depending on the adjuvant, humoral or cellular immunity is selectively stimulated. T-helper lymphocytes and cytotoxic lymphocytes (CTL) can be activated. In addition, modulation of the immune response can be directed toward MHC I or MHC II. Th1 or Th2 responses can be preferentially activated, resulting in the secretion of corresponding cytokines. Finally, they modulate antibody avidity and specificity [71].

Immunostimulatory adjuvants can mimic alarmins that target specific receptors in the innate immune system, such as the molecular pattern recognition receptors (PRRs) that activate APC cells. The latter modulate their phagocytic activity and optimize antigen presentation and cytokine secretion according to the PRRs stimulated [77,78,90].

Antigen bioavailability can be considerably prolonged by delaying its release through various means, including activation of the innate immune system, for example by creating inflammatory foci locally or within lymph nodes to stimulate immune memory, and by promoting local recruitment of immune cells [91]. According to Ramon, the local inflammation increased via the action of tapioca would retain the antigen at the injection site and gradually release it, promoting antibody production [18,76]. It is likely that the immunological mechanism is more complex and involves, at least in part, a non-specific, adaptive immune response such as the release of immune effectors [69].

In recent years, research has focused on Toll-like receptor (TLR) ligands, whose activation triggers a strong and immediate innate immune response, followed by an adaptive immune response. Among the TLR agonists, two are used in licensed vaccines. Monophosphoryl lipid A (MPL), which activates TLR4, is used in the AS04 and AS01 adjuvants. CpG-1018 is a TLR9 agonist and adjuvant for a hepatitis B vaccine approved in 2017 [92]. 

The adjuvant can also protect antigens from degradation by the body’s own enzymes.

Finally, some delivery system adjuvants target APCs by adsorbing many molecules of the same antigen to induce a stronger adaptive immune response, or by delivering the antigen to an immune-privileged site, such as lymph nodes or the M cells of Peyer’s patches, to induce mucosal immune responses [81].

**Table 1:** Main adjuvants and their effects on immune response (Th1/Th2 = type 1 T helper/type 2 T helper; APC = antigen-presenting cell; PS = polysaccharide; TLR = toll-like receptor; HA = hemagglutinin; MHC = major histocompatibility complex; AAHS = amorphous aluminum hydroxyphosphate sulfate; MPLA = monophosphoryl lipid A; QS-21 = saponin from *Quillaja saponaria*; TLR = Toll-like receptor).

Adjuvant	Discovery (D) or Registered (R) Dates	Mode of Action	Use in Human	References
Tapioca	1925 (D)	Depot effect	No	[76]
Aluminum-containing adjuvants: Aluminum hydroxide (AlOOH), AAHS	Alum: 1926 (D) AlOOH: 1938 (D) AAHS: 2006 (R)	Depot effect; High Th2 response; Stimulation of the innate immune response; APC recruitment and activation; Optimization of APC phagocytosis	AlOOH: DTP Pertussis; Poliomyelitis; Pneumococcus; Hepatitis A; Hepatitis B; Anthrax AAHS: *Hemophilus*-type B; Hepatitis A (inactivated); Hepatitis B (recombinant); Tetravalent Papillomavirus (recombinant)	[51,93,94]
Glycoconjugation (PS + toxoid)	1929 (D)–1985 (R)	PS = Increased production of specific antibodies; Toxoid = stimulation of immune memory	*Hemophilus*-type B; Meningitis AC; Pneumococcus 13	[95,96]
Calcium salts	1931 (D)–1960 (R)	Depot effect; Stimulation of cell immunity; Increased production of specific antibodies; Th1 and Th2 responses	DTP Pertussis; Poliomyelitis	[97,98]
Freund’s adjuvant (water-in-oil emulsion)	1939 (D)	Depot effect; Stimulation of the innate immune response; Th1 response	No (except incomplete Freund’s adjuvant)	[99]
Saponin	1947 (D)	High Th1 and Th2 responses	Used in combination in adjuvant platforms	[90,100,101,102]
Lipopolysaccharides (LPS, MPLA)	1956 (D)	Activation of TLR4	Used in combination in adjuvant platforms	[103]
Liposomes	1974 (D)	Depot effect; Optimization of APC phagocytosis; Increased production of specific antibodies; Stimulation of cell immunity; Moderate Th2 response	Hepatitis A; Papillomavirus; Influenza; Malaria	[104,105,106]
Virosomes	1975 (D)–1994 (R)	HA-mediated cytoplasmic antigen delivery; APC recruitment and activation; Optimization of APC phagocytosis; Cytokine production	Influenza; Hepatitis A	[106,107,108]
Cytokines	1983 (D)–19xx (R)	Depending on cytokines: Enhancement of other cytokine production; Increases MHC-II expression on APC; APC recruitment and activation; Stimulation of immunological memory	Licensed for therapeutic vaccines against skin cancers and autoimmune skin disorders; Experimental studies with Influenza, Hepatitis B, Malaria and Flaviviridae vaccine candidates; Not yet approved for infectious disease human vaccines	[73,109,110,111,112]
Nucleic acid-based (dsRNA, Poly(I:C), CpG 1018)	1988 (D)–2017 (R)	Activation of TLR3 (dsDNA, Poly(I:C)) or TLR9 (CpG 1018); APC recruitment and activation; Optimization of APC phagocytosis; Th1 response	Hepatitis B (recombinant); SARS-CoV-2 spike (recombinant)	[113,114,115,116]
MF-59 (oil-in-water emulsion)	1997 (R)	Depot effect; APC recruitment and activation; Optimization of APC phagocytosis; Th1 and Th2 responses	Influenza (inactivated)	[117]
AS04 (MPLA + alum)	2004 (R)	APC recruitment and activation; Activation of TLR 4; Enhancement of cytokine production;	Hepatitis B; Papillomavirus bivalent (recombinant)	[113,118]
AS03 (oil-in-water emulsion)	2009 (R)	Depot effect; Stimulation of the innate immune response; APC recruitment and activation; Optimization of antigen presentation to the lymphatic system	Influenza H5N1 (inactivated)	[119]
AS01 (MPLA + QS-21)	2015 (R)	Stimulation of the innate immune response; Recruitment and activation of leukocytes and APC; Activation of TLR 4; Optimization of antigen presentation to the lymphatic system	Malaria; Varicella-Zoster (recombinant)	[113,120]
Lipid nanoparticles	2020 (R)	Depot effect; Sustained antigen release; Protection and bioavailability of antigens; Optimization of antigen presentation to the lymphatic system	COVID-19	[78]

## 6. Fourth Discovery: Combined Vaccination (1926)

For Ramon, the combination of antigens follows directly from the principle of adjuvants and stimulants. However, to gain acceptance for multiple vaccine combinations, Ramon had to battle against the idea of “multiple antigen competition” that prevailed in his day. At the time, it was thought that the addition of antigens proportionally reduced the immune response capacity of the organism. Ramon set out to show that the combination of antigens not only did not exhaust the immune system, but stimulated it. The combination of diphtheria (or tetanus) toxoid with the anti-typhoid and paratyphoid TAB vaccines was as well tolerated as the administration of either vaccine alone, and the immune response was superior, almost as high as with the addition of an adjuvant—at least in the case of tetanus and diphtheria, since the protective power of the TAB vaccine could not be measured. The two vaccines available at the time, heated TAB and lipo TAB, were used with the same results [19,65,97]. However, by the end of World War II, the effectiveness of combined vaccination against diphtheria, tetanus, typhoid and paratyphoid fevers could be measured by the substantial reduction in morbidity and mortality from typhoid or paratyphoid fever compared with the period when TAB was administered alone [121].

In addition to the benefits of boosting cellular and humoral immunity to associated antigens, the logistical and economic advantages of multiple vaccinations with known benefit/risk profiles are now well established. For children and adolescents, combined immunization against tetanus, diphtheria, pertussis, meningococcal disease and human papillomavirus is recommended. Influenza, pneumococcal and herpes zoster vaccines are recommended for the elderly and special risk groups. Similarly, multiple vaccines may be combined for travelers [19,122].

## 7. Conclusions

Ramon was the most nominated scientist for the Nobel Prize in Medicine—155 times between 1930 and 1953, the last year for which this information was published by the Nobel Foundation [123].

Ramon’s work has proved particularly productive. In terms of the impact on immunization and public health, the practical consequences of the use of toxoids, adjuvants and associated vaccinations are considerable, if we consider only the benefits of diphtheria and tetanus immunizations, not to mention veterinary vaccinations. In addition, Ramon’s research has opened new avenues of investigation, for example in the field of adjuvants, with particularly fruitful practical and conceptual results in terms of basic immunology.

His discoveries were not accidental. They were the result of careful observation of phenomena occurring during the immunization of animals used to produce therapeutic serums. The state of knowledge at the time prepared these discoveries, which led to disputes over priority. Some scientists claimed authorship of Ramon’s discoveries, although they had merely observed a phenomenon that Ramon was able to reproduce, explain and develop [124,125]. Ramon was also able to use the observations he made, such as the antiseptic and detoxifying effect of formalin, to respond to constraints or limitations. However, unlike the other scientists who carried out these operations, Ramon sought the causes and skillfully exploited the consequences he had carefully recorded and interpreted, opening the way to new discoveries.
